# Ligand Docking to Intermediate and Close-To-Bound Conformers Generated by an Elastic Network Model Based Algorithm for Highly Flexible Proteins

**DOI:** 10.1371/journal.pone.0158063

**Published:** 2016-06-27

**Authors:** Zeynep Kurkcuoglu, Pemra Doruker

**Affiliations:** Department of Chemical Engineering and Polymer Research Center, Bogazici University, Bebek, Istanbul, 34342, Turkey; University of Minnesota, UNITED STATES

## Abstract

Incorporating receptor flexibility in small ligand-protein docking still poses a challenge for proteins undergoing large conformational changes. In the absence of bound structures, sampling conformers that are accessible by apo state may facilitate docking and drug design studies. For this aim, we developed an unbiased conformational search algorithm, by integrating global modes from elastic network model, clustering and energy minimization with implicit solvation. Our dataset consists of five diverse proteins with apo to complex RMSDs 4.7–15 Å. Applying this iterative algorithm on apo structures, conformers close to the bound-state (RMSD 1.4–3.8 Å), as well as the intermediate states were generated. Dockings to a sequence of conformers consisting of a closed structure and its “parents” up to the apo were performed to compare binding poses on different states of the receptor. For two periplasmic binding proteins and biotin carboxylase that exhibit hinge-type closure of two dynamics domains, the best pose was obtained for the conformer closest to the bound structure (ligand RMSDs 1.5–2 Å). In contrast, the best pose for adenylate kinase corresponded to an intermediate state with partially closed LID domain and open NMP domain, in line with recent studies (ligand RMSD 2.9 Å). The docking of a helical peptide to calmodulin was the most challenging case due to the complexity of its 15 Å transition, for which a two-stage procedure was necessary. The technique was first applied on the extended calmodulin to generate intermediate conformers; then peptide docking and a second generation stage on the complex were performed, which in turn yielded a final peptide RMSD of 2.9 Å. Our algorithm is effective in producing conformational states based on the apo state. This study underlines the importance of such intermediate states for ligand docking to proteins undergoing large transitions.

## Introduction

Proteins undergo conformational changes of varying degrees, in order to perform their cellular functions. The flexible nature of proteins is one of the challenging problems in drug design studies since even small conformational changes can affect the nature of ligand-protein interactions [1]. In recent years, there has been an increasing effort for addressing this issue in docking algorithms. The strategies for incorporating protein flexibility include limited conformational flexibility for side chains; conformational generation on the fly and ensemble docking, which in turn improve the docking performance compared to rigid docking [2]. However, accounting for large conformational changes upon binding is still challenging [3] and efficient computational algorithms are necessary to sample protein conformations for more accurate prediction of binding sites and affinities in docking studies.

Elastic network model (ENM) [4–10] is a coarse-grained normal mode approach employing pair-wise harmonic interactions. It is a computationally efficient tool that can provide insight about the protein functional dynamics and conformational changes that take place upon ligand binding. ENM has been widely used in protein-protein docking to account for the global backbone conformational changes [3,11–13], as well as in the optimization of complexes fitted in electron-density maps and in protein-DNA model refinement [13,14].

ENM has also gained recognition in drug design studies for incorporating receptor backbone flexibility in small ligand-protein docking. In an earlier study [15], deformation along a combination of so-called “relevant” normal modes acting on the binding region has produced an ensemble of cAMP-dependent protein kinase conformations, which in turn increased the docking accuracy of ligands. In another study, application of ENM on heavy atoms of the residues within a specific cutoff from the cognate ligand for generating multiple receptor conformations has improved the docking results on 28 proteins [16]. However, Dietzen et al. [17] have shown that ENM does not significantly improve the docking performance when local ligand-specific induced fit movements and “binding pocket restricted” normal modes are used for reconstructing the holo structures of 433 proteins from apo structures. Bolia et al. [18] introduced BP-Dock for both receptor backbone and side-chains flexibility, based on perturbation response scanning [19], ENM and linear response theory. The technique was applied on a large dataset of unbound structures with unbound/bound RMSD varying from 0.103 to 1.65 Å, to generate various binding site conformations even in the absence of ligand.

Apart from concentrating on the modes related only with the binding pocket, May and Zacharias [20] have included receptor global flexibility by relaxation along 10 softest ENM modes, which has improved the results of inhibitor-kinase (CDK2) docking and cross-docking experiments compared to the rigid receptor case. One should note that all above mentioned ENM-based studies in ligand docking focus on proteins with relatively local conformational changes.

As for proteins undergoing large conformational changes, two approaches have been used for holo structure prediction, namely tCONCORD [21,22] and the “conformation explorer”[23]. The tCONCORD ensemble is generated based on the geometrical description of a given protein structure and biased by imposing a shape constraint for the holo structure. For 8 out of 10 proteins (with domain closure of 2–7 Å), close-to-native poses exist among the top-ranked models. The conformation explorer algorithm which employs a multistage, induced-fit approach for hinge-bending proteins, has been applied to five single-chain proteins composed of two domains. Details on these studies will be provided at the end of results section, together with a comparison to our current method.

Even though ENM is widely used for exploring the biased and unbiased transitions of proteins including large conformations changes [24–38], the utility of resulting conformers in ligand docking has not been addressed so far, at least to our knowledge. Our current study presents an unbiased atomistic conformer generation algorithm by integration of ENM with energy minimization and clustering, which is especially beneficial for proteins undergoing large conformational transitions. Our algorithm uses only the apo crystal structure as input and iteratively produces conformers that are accessible from the apo state. Application of the technique to adenylate kinase (AK), two periplasmic binding proteins (LAO and DBP), homo-dimeric biotin carboxylase (BC) and calmodulin (CAM) will be considered first in terms of holo structure prediction. Then, the efficacy of generated conformers in ligand docking will be presented for the same dataset by ensemble docking to apo, intermediate and close-to-holo states. In particular, calmodulin is the most challenging case, expressing a rearrangement of 15 Å RMSD between its apo state and complex with a 19-residue long helical peptide.

## Results

### Overview of dataset

**Fig 1:**
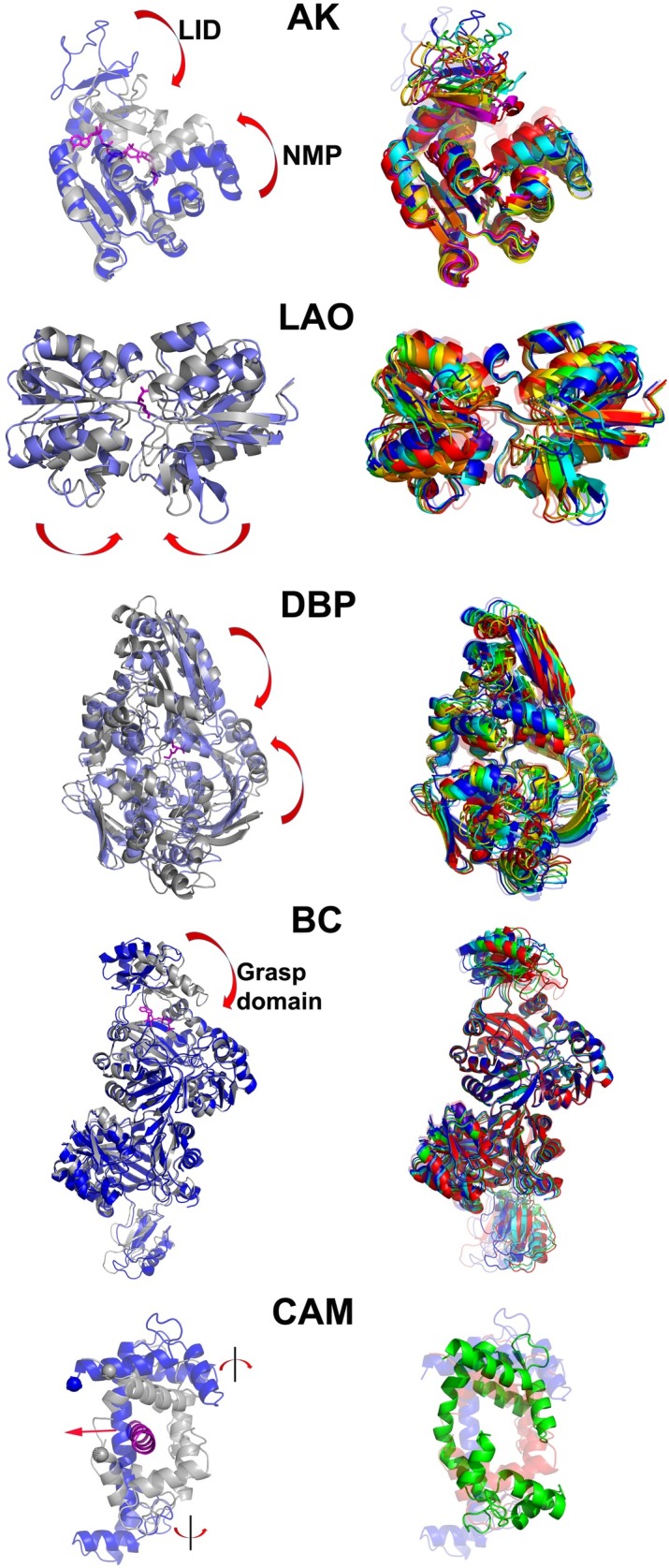
Conformational changes of the proteins in our dataset. Left panels show the starting open conformer (blue) that is aligned on the holo structure (grey) with the bound ligand in magenta sticks (cartoon in CAM). For all cases except CAM, the generated conformers that are later used in docking are shown in the right panels aligned on open (transparent blue) and closed (transparent red) structures. For CAM, green conformer is the conformer that has minimum RMSD to the holo structure, generated from CAM simulation. This conformer is used in peptide docking, and the resulting docked structure is taken as the starting point for CAM-pep simulation.

We applied the new atomistic conformer generation algorithm to five proteins listed in Table 1. These proteins represent a diverse set in terms of size, topology and dynamic domain decomposition. Large conformational changes (RMSD > 4 Å) between unbound and bound forms of receptor are detected in all cases, and these are shown in the left panels of Fig 1 (arrows indicate the domain motions). Specifically, monomeric LAO and DBP exhibit hinge-bending closure of two domains with the ligand binding in between them. AK is composed of three dynamic domains that enclose its ligands. In homo-dimeric BC, each monomer undergoes a hinge-type closure of its small domain over the large core. Calmodulin, the smallest protein in our set, presents the most challenging case in terms of domain motion. The dumbbell-shaped CAM in its extended state exhibits both a bending of its central long helical backbone and twisting of the two lobes connected to it during peptide binding. The overall RMSD is extremely large (15 Å) between extended and fully closed CAM structures.

**Table 1:** Proteins used in conformer generation and ligand docking.

Protein	Number of residues	PDB id of the apo structure	RMSD between apo and complex (Å)	PDB id of the complex	Ligand in complex
Adenylate kinase (AK)	214	4ake [39]	7.2	1ake [40]	Bis(adenosine)5’-pentaphosphate (AP5)
Lysine-arginine- ornithine-binding protein (LAO)	238	2lao [41]	4.7	1lst [41]	Lysine
Dipeptide-binding protein (DBP)	507	1dpe [42]	6.5	1dpp [43]	Glycyl-leucine
Biotin carboxylase (BC)	894	1dv1 [44]	4.6/ 4.1^*a*^	1dv2 [44]	Adenosine triphosphate (ATP)
Calmodulin (CAM)	144	1cll [45]	15	1wrz [46]	19-residue long helical peptide

^*a*^ First and second RMSDs are for monomers A and B, respectively. Overall RMSD is 4.5 Å for dimeric BC.

We further performed ligand docking on a sequence of generated conformers, which are shown on the right panels of Fig 1 for each protein. The specific ligands listed in Table 1 (shown in Fig 1) are also quite diverse. In the special case of CAM, the peptide is docked to the green structure shown on the right panel, and the conformer generation procedure is carried on the complex.

The missing loops in the crystal structure 1dv1 (residues 162–168 and 190–194 in chain A, 160–168 and 188–198 in chain B) were modelled using ModLoop [47,48] before conformer generation.

### Evaluation of conformer generation procedure

#### Blind search

The iterative algorithm performs ENM on the energetically-minimized apo structure and deforms the structure along all combinations of the first three collective modes using a fixed RMSD (3 Å for the largest conformational change of CAM and 2 Å for others). The resulting conformers are clustered to reduce the redundancy. A representative conformer is selected from each cluster if it is distinct from the parent cluster(s). Selected conformers are further subject to energy minimization using implicit solvent, in order to correct unrealistic distortions in the structure. Such a set of energetically minimized conformers constitutes a “generation”. In the next generation, ENM and deformation steps are performed on each new (minimized) cluster representative. In this ‘blind’ search procedure, no bias is imposed towards a target or bound/complex structure. The details of the generation procedure are given in Methods.

Table 2 summarizes the total number of distinct conformers (clusters) including all generations produced for each protein. RMSD values for the receptor are calculated based on alpha carbons. Several conformers (4 to 7) were found close to the bound structure (RMSD < 3 Å) among the conformers generated at the end of blind search for each protein except CAM. Minimum RMSD values are within the range of 1.4–2.8 Å for cases except CAM. CAM’s initial open-to-closed RMSD of 15 Å decreases to 6.8 Å during 8 generations, but full domain-closure present in its complex could not be realized. However, in a second conformer generation procedure (CAM-pep) performed in the presence of a docked peptide, the final minimum RMSD of 3.7 Å of complex is attained after three cycles. This issue will be discussed in detail in CAM docking results.

**Table 2:** Conformer generation and docking results.

Protein	No. of gens	Blind conformer generation			Energy-based conf. generation			Docking
		No. of conf. ^*a*^	No. of conf. (< 3 Å)^*b*^	Min. RMSD^*c*^ (Å)	No. of conf.^*a*^	No. of conf. (< 3 Å)^*b*^	Min. RMSD^*c*^ (Å)	Ligand RMSD^*d*^ (Å)
AK	7	185	6	2.8	92	4	2.4	2.9
LAO	7	84	7	2.2	8	4	1.7	2.0
DBP	5	37	4	1.4	-	-	-	1.5
BC	4	22	5	1.7^*b*^	22	5	1.7	1.8
CAM	8	451	0	6.8	106	0	9.2	-
CAM-pep	3	16	0	4.0	11	0	3.8	2.9

^*a*^ Total number of conformers after RG filter is reported here. Original number of conformers before filtering are provided in S2–S10 Tables together with details on individual generations.

^*b*^ Number of conformers that are close to the holo structure within 3 Å RMSD is given.

^*c*^ Minimum RMSD to holo structure based on alpha carbons is reported. For homo-dimeric BC, the minimum value is observed in subunit B. The value for subunit A is 2.3 Å.

^*d*^ Minimum ligand RMSD in the docking ensemble is reported. Ligand RMSD is calculated based on all heavy atoms of AP5, Lys, Glycil-leucine, ATP and on backbone atoms of 19-res peptide. For AK, LAO and DBP, these values also correspond to the clusters with the most favorable score and largest number of elements.

#### Energy-based search

We imposed an additional criterion during conformer generation based on the energy of the apo structure, which is obtained from minimization in implicit solvent. This was inspired by the free energy map for AK [49], which has indicated various conformational states of the protein with lower energy than the open structure, including the closed conformer. In our “energy-based” search, clusters/conformers having higher energy than the apo state are not allowed to be parents for the subsequent generation. If no conformers with lower energy than the apo state exist at the end of a generation, the procedure ends. As a result, conformers with lower energy than the apo state are emphasized in terms of sampling.

This procedure significantly reduces the number of generated conformers for AK, LAO and CAM (Table 2), which have lower energy in the holo state (without ligand) compared to the apo state (see S1 Table). The procedure was not applicable for DBP, because all conformers produced in the first generation had higher energies than apo. In the case of BC, it did not lead to reduction in the number of generated conformers. Thus, energy-based selection, if applicable to the specific protein at hand, can reduce the number of conformers considerably and provide a closer approach to bound structure as observed for AK and LAO.

#### RG filtering

**Fig 2:**
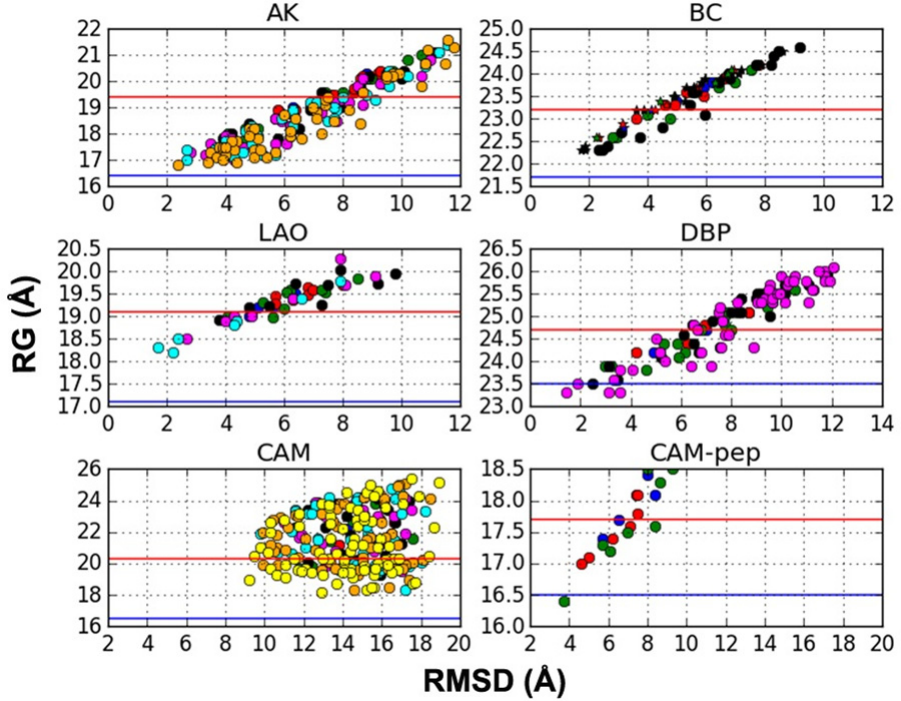
Relation between radius of gyration and RMSD (based on alpha carbons) to bound structure, where the conformers are colored according to generation. (a) AK (energy-based), (b) BC (blind/energy-based, stars and circles belong to different chains), (c) LAO (energy-based), (d) DBP (blind), (e) CAM energy-based and (d) CAM-pep (energy-based). Red and blue horizontal lines refer to the monomeric RG of the apo and holo crystal structures, respectively. The conformers above the red line are discarded during RG post-filtering.

Fig 2 shows the correlation between radius of gyration (RG) and RMSD to the holo structure for all generations/conformers. Here conformers from gen1 to gen8 are distinguished by filled circles of different colors (in the respective sequence of blue, red, green, black, magenta, cyan, orange and yellow). As the chain gets more compact, i.e. its RG is reduced, conformers get closer to bound structure, which is stabilized in the presence of ligand. Such correlations have proven useful for the prediction of bound structures [24,34,50] for hinge-bending type transitions by imposing constraints on RG. The only exception is apo CAM, which undergoes a more complex transition (Fig 1) including bending of the central helix and twisting of the lobes. Interestingly, binding of the peptide induces further closure of CAM-pep.

Here we do not impose any constraints during conformer generation, but RG is rather used as a post-filtering criterion to discard conformers with higher RGs than the apo structure. As a result, the total number of conformers generated by blind or energy-based search are significantly reduced (by 25–70%). In Table 2 the total number of conformers remaining after RG filter is reported as a summation over all generations. S2–S10 Tables provide details on the number of distinct conformers in each generation (before and after RG filter) and a classification according to their backbone RMSD to the holo structure.

#### Computational efficiency of the method

The computational time required for conformer generation depends on system size (total residue number) and number of generations/cycles. For example, the 7^th^ cycle of AK (with 214 residues) producing 149 conformers in blind search completes in 1 hour using 6 nodes, where each node holds 2 Intel Xeon X5670 CPUs with 6 cores. Most of this time is spent for the minimization of the structures using implicit solvent model. Relaxing the convergence criterion in minimization reduces the computational time. Thus, the application of the technique to supramolecules becomes computationally feasible (results in preparation).

### Ligand docking to generated conformers

Discarding conformers with RGs larger than the apo state is reasonable if one is interested in close-to-holo or intermediate states, i.e. between open and closed forms. Our strategy is to start docking with the conformer having the lowest RG value, which lies close to the bound state. We then perform dockings to its parent conformers going up to the apo structure. Fig 1 (right panels) shows this sequence of conformers in rainbow colors, where blue and cyan are relatively open conformers (from gen1 and gen2); green, yellow, orange and magenta correspond to gen3 and up, and the conformer from final generation is always red. In what follows, we will separately discuss docking results for each entity in our dataset.

#### Adenylate kinase

AK is a phosphotransferase enzyme that plays an important role in cellular energy homeostasis. It catalyzes the conversion of ATP and AMP into two ADP molecules within the cells. AK is composed of three domains, namely the CORE, the LID (ATP-binding) and the NMP (AMP-binding) domains. In Fig 1, the LID and NMP domains undergo large conformational changes (about 7 Å) around the CORE domain to provide a solvent-free environment for substrates. Both experimental and computational studies [51,52] suggested that the closed state of AK is accessible even in the absence of ligand.

We performed dockings on a sequence of AK conformers (gen7, gen6,.., gen1, apo), which comprise the lowest RG conformer from the 7^th^ cycle of the energy-based search and its parents. The ligand is a large inhibitor AP5 that mimics the substrates AMP and ATP together. In Table 3, the ligand RMSD with respect to its positioning in the crystal structure (1ake) is reported for the first cluster, which has the highest score and the largest population at the same time for each conformer.

**Table 3:** Docking results for AK conformers.

Conformer	Backbone RMSD^*a*^ (Å)	Ligand RMSD^*b*^ (Å)	Binding energy^*c*^ (kcal/mol)	Number of poses ^*d*^	Number of clusters
**complex (1ake)**	**0.0**	**0.6**	**-15.96**	**100**	**1**
apo (4ake)	7.1	7.4	-2.96	100	1
gen1	6.3	7.8	-2.96	100	1
gen2	5.0	4.8	-2.49	98	2
gen3	4.1	3.4	-3.97	100	1
**gen4**	**4.2**	**2.9**	**-7.06**	**100**	**1**
gen5	3.5	6.5	-1.23	100	1
gen6	2.7	15.6	-3.24	45	6
gen7	2.4	5.1	-2.71	95	2

^*a*^ Receptor RMSD is calculated with respect to the 1ake.

^*b*^ Ligand RMSD is calculated with respect to AP5 in the complex (1ake).

^*c*^ Binding energy and number of elements/poses are given for the first cluster that has the highest score from AutoDock v4.

^*d*^ Total number of docked poses is equal to 100.

**Fig 3:**
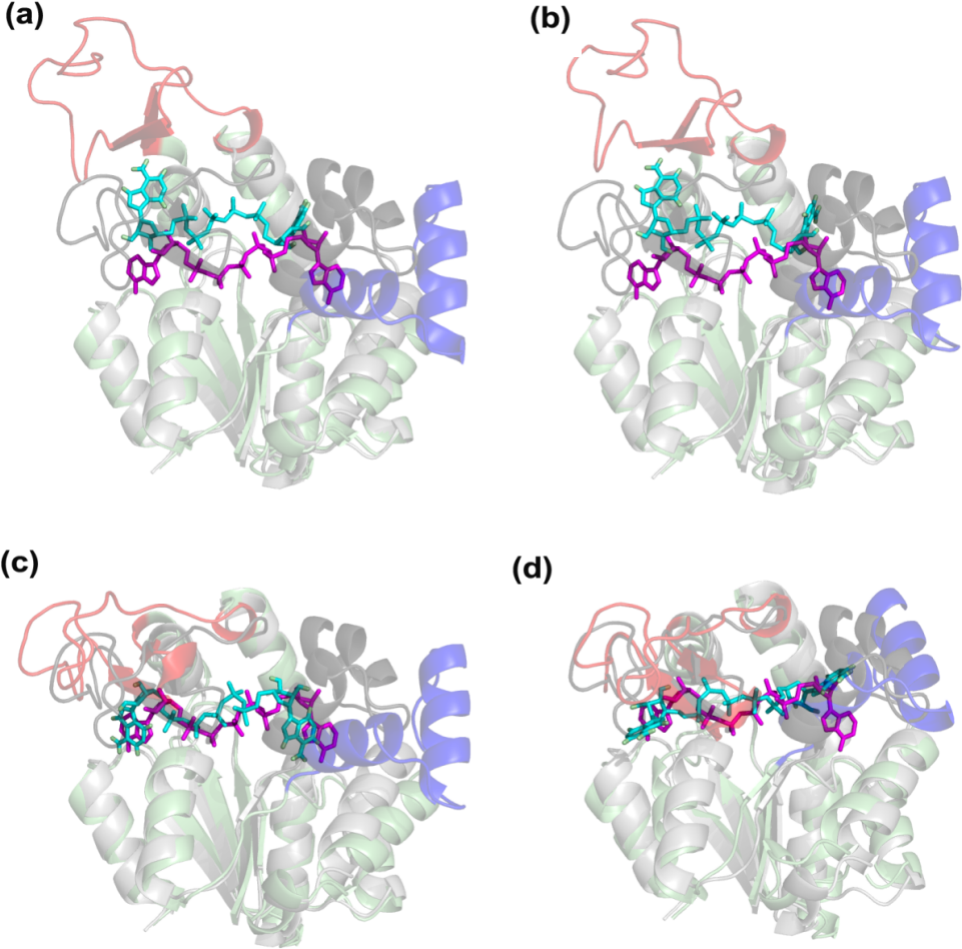
AK docking poses are shown for (a) apo, (b) gen1, (c) gen4, (d) gen7, where LID, core and NMP domains are red, green and blue, respectively. Each pose is aligned onto the closed crystal structure (gray, NMP and LID are colored black). The ligands in the closed crystal structure and docking pose are shown as magenta and cyan sticks, respectively. Interestingly, the best overlapping docking pose with the crystal structure corresponds to gen4 corresponding to an intermediate state with partially closed LID and open NMP.

Fig 3 shows the docking poses for apo, gen1, gen4 and gen7. The conformer gen7, which lies closest to the bound receptor structure (2.4 Å RMSD), has a ligand RMSD of 5 Å. However, the highest docking score is obtained for gen4, which presents a pose with satisfactory ligand RMSD of 2.9 Å. As opposed to the complex with fully closed LID and NMP domains, gen4 presents an ‘in-between’ state, where the LID is partially closed and the NMP is open. Interestingly, such a state conforms with a highly populated intermediate state observed in AMP/ATP binding to AK, both in experimental and computational studies [53,54], and also similar to a crystal structure with pdb id 1dvr [55]. When we repeat the dockings with other conformers similar to gen4 and gen7, we obtain the same trend of preference for the intermediate state (not shown here).

S11 Table provides inhibitor-receptor interaction details, where 19 residues interacting with the ligand (within a cutoff of 4.5 Å) are observed in the crystal structure 1ake. Among the generated conformers, gen4 presents the highest number of residues interacting the inhibitor (10 residues). Eight of these residues are common with those in the crystal, which are located on the CORE (G10, G12, K13, G14, R167 and K200), the LID (R123) and the NMP (R36) domains.

#### Biotin carboxylase

In fatty acid synthesis, BC catalyzes ATP-dependent carboxylation of biotin. It has a homo-dimeric structure with each subunit having its own catalytic site. In each subunit the ATP grasp domain closes over the globular core, as a result of which each subunit undergoes a conformational change of 4 Å. Experimental evidence suggest that two subunits alternate their catalytic cycles, exhibiting half-sites reactivity [56].

In generated conformers (Fig 1), we do not observe simultaneous closure of grasp domains on different subunits, which is in fact consistent with experimental observation [56]. So, application of the RG criterion independently for each monomer (A or B) is suitable for BC. Selected conformers together with the apo and closed structures clearly exhibit the closure of the ATP grasp domain (subunit B is emphasized in Fig 1).

**Fig 4:**
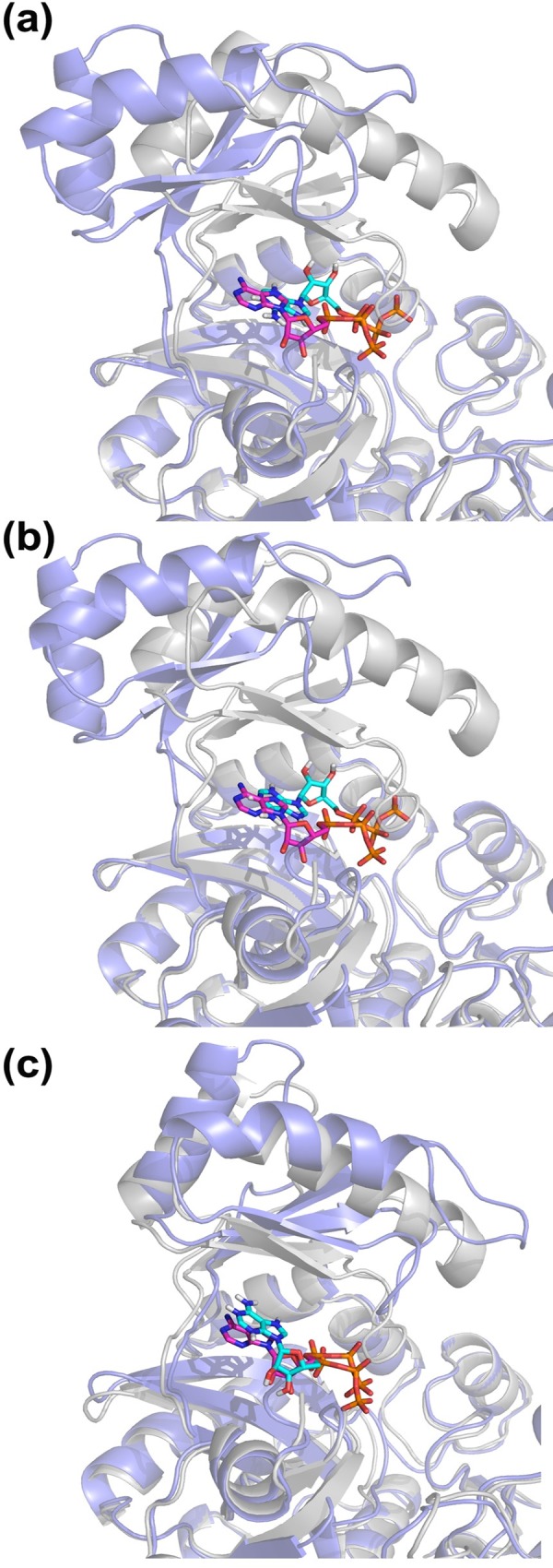
BC docking poses (blue) for (a) apo cluster 4, (b) gen1 cluster3, and (c) gen4 cluster 1, (aligned on ligand-bound complex (1dv2, grey). Ligand positioning in crystal and docked structures is shown in magenta and cyan stick representation, respectively. The docking poses are shown for lowest ligand RMSD cluster in dockings (details are given in S12 Table). The best pose corresponds to the conformer closest to the bound crystal structure (gen4). There are seven common residues with those in the crystal, surrounding the ligand at the binding site (out of eleven). The list of interacting residues is given in S13 Table.

Docking poses (Fig 4) indicate that as the ATP grasp domain closes over the globular section, ATP positioning gets closer to bound structure. The best ligand RMSD is 1.8 Å in gen4 dockings, which belongs to the cluster with highest score (second most populated cluster, details given in S12 Table). It should be noted that there is E288K mutation at the active site that stabilizes the specific ATP-BC complex (pdb id: 1dv2) in the absence of two Mg ions that exist in the active enzyme. Although we generated conformers starting from wild type apo structure (pdb id: 1dv1), we incorporated this mutation in the conformers used in ATP docking.

#### Periplasmic binding proteins (PBP)

We will collectively present the results for two PBPs, namely LAO and DBP, under this section. PBPs serve to transport a wide variety of sugars, amino acids, peptides and inorganic ions into bacteria. They consist of two domains connected by a hinge region at the interface, where the ligand binding site is located. Large bending motion around a hinge facilitates the alternation between open/apo and closed/ligand-bound conformations [57]. Specifically, LAO and DBP exhibit open-to-closed structural rearrangements amounting to 4.7 Å and 6.5 Å, respectively.

**Fig 5:**
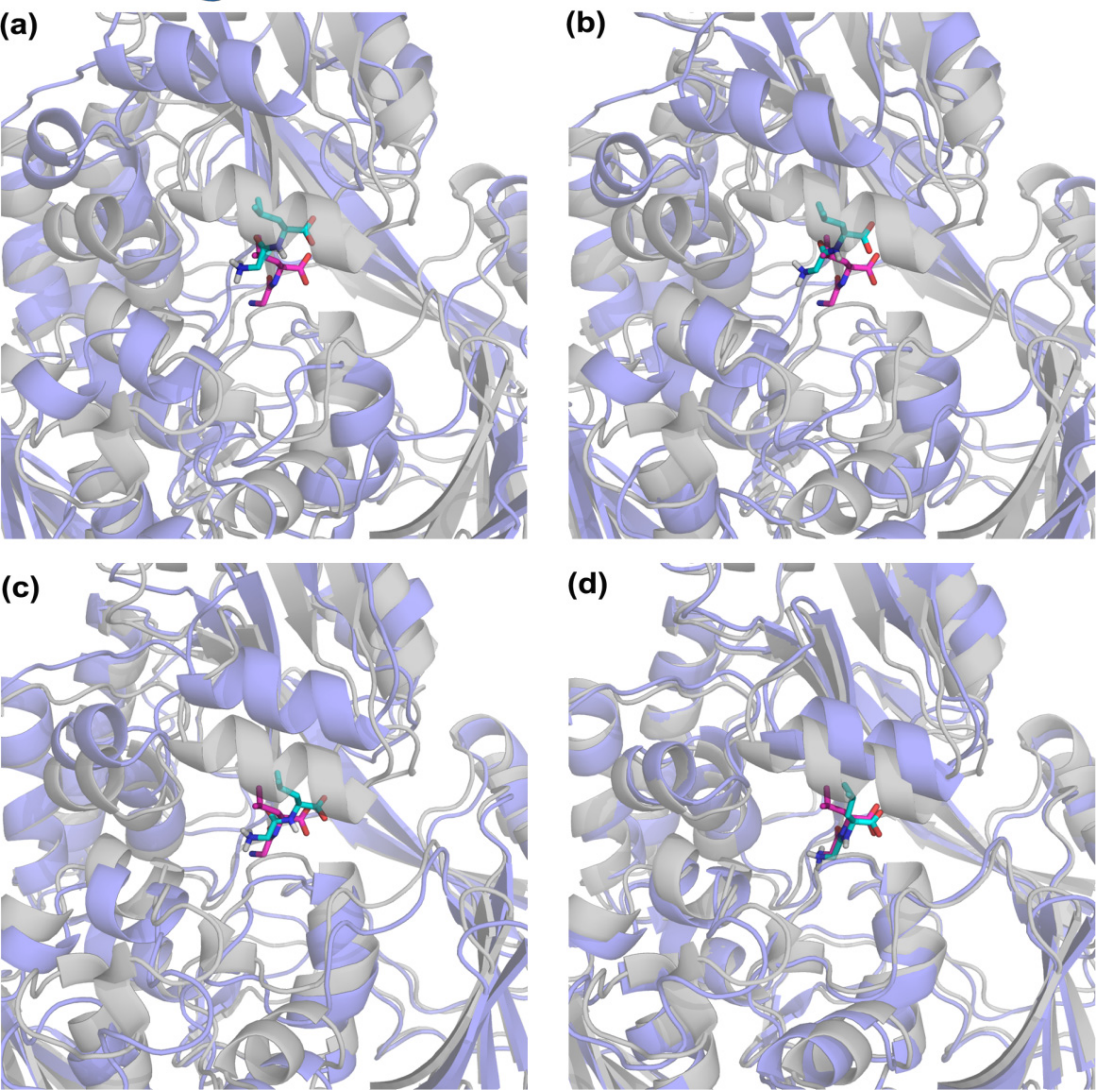
DBP docking poses (blue) for (a) apo, (b) gen1, (c) gen3 and (d) gen5 aligned to the bound crystal structure (gray). Ligands in crystal and the docking poses are shown in magenta and cyan stick representation, respectively. The best pose corresponds to the conformer closest to the bound crystal structure (gen5).

Dockings of the ligand Gly-Leu were performed on the lowest RG conformer of DBP from the last cycle of blind search and its parents (see Fig 5 and Table 4). Docking to gen5 conformer, which is closest to the closed crystal structure, yields the highest score and the lowest ligand RMSD (1.5 Å). So peptide binding to DBP seems to be in line with full domain closure. Still, correct positioning of the ligand and part of the ligand-receptor interactions (S14 Table) are even present in apo and intermediate state dockings. Therefore, induced-fit type of domain closure could also take place as an alternative to selection of the closed state.

**Table 4:** Docking results for DBP conformers.

Conformer	Backbone RMSD (Å) to complex	Ligand RMSD (Å)	HADDOCK score^*a*^	Numberof elements ^*a*^	Numberof clusters
**complex (1dpp)**	**0.0**	**0.5**	**-32.7±1.7**	**153**	**5**
apo (1dpe)	6.5	6.1	-19.1±3.3	195	1
gen1	4.9	4.9	-19.1±1.5	199	1
gen2	4.2	3.8	-21.0±1.3	198	1
gen3	3.0	2.2	-20.2±1.9	198	1
gen4	2.5	3.2	-24.9±1.6	200	1
**gen5**	**1.4**	**1.5**	**-31.4±2.6**	**193**	**2**

^*a*^ HADDOCK score and number of elements is only given for the cluster having the highest score. Total number of clustered poses is equal to 200.

**Fig 6:**
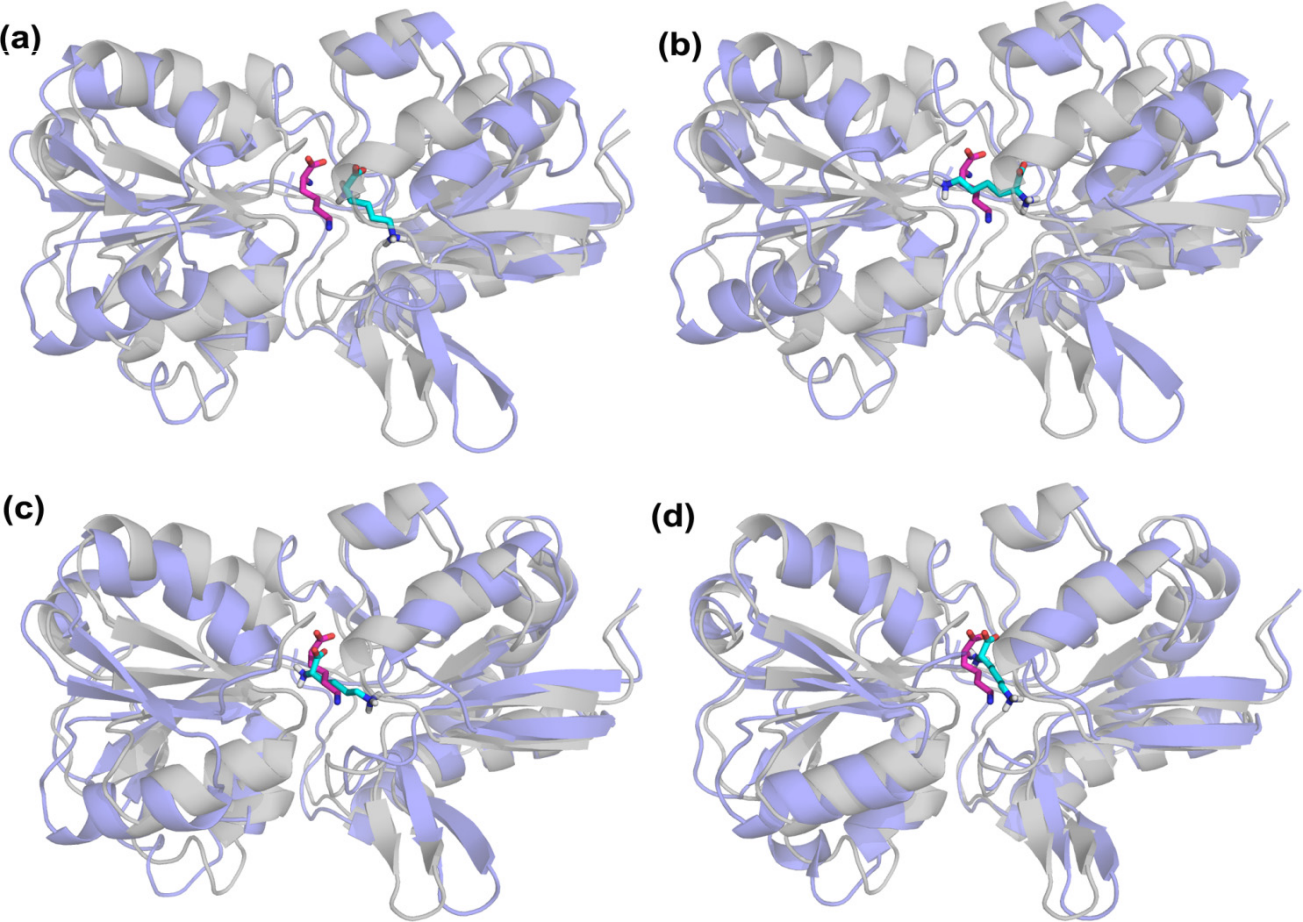
LAO docking poses are shown (purple) for (a) apo, (b) gen1, (c) gen4 and (d) gen6 aligned to the closed crystal structure (gray). Ligands in crystal and the docking poses are shown in magenta and cyan stick representation, respectively. Like DBP, the best pose with the highest score is obtained for gen6 that is closest to the holo structure.

Lys dockings on LAO binding protein were performed using conformers from energy-based search (see S14 Table for details). In the specific sequence of conformers, relatively open conformers (apo, gen1 to gen3) yield eight clusters and relatively high ligand RMSDs. As the conformers get closer to the closed structure (gen5 and gen6 with respective backbone RMSDs of 2.7 and 1.7 Å), highest scores and low ligand RMSDs (~ 2 Å) are observed for the most populated clusters. The total number of clusters for gen4-gen6 are considerably less compared to the rest (apo and gen1-gen3). Specifically, a favorable binding mode is observed for gen4, which is in fact an intermediate state with a backbone RMSD of 3.8 Å. Some of these conformers and docking poses are shown in Fig 6.

For both periplasmic binding proteins that undergo hinge-bending closure of two domains, better docking scores and ligand positioning are obtained as the conformer gets closer to the holo form. Thus, the ligand seems to favorably bind to the closed state (9/9 and 5/9 residues in common with crystal for DBP and LAO, respectively. See S14 and S16 Tables). Yet, our results also hint to the possibility of ligand binding to intermediate states followed by an induced-fit type of domain closure.

#### Calmodulin

**Fig 7:**
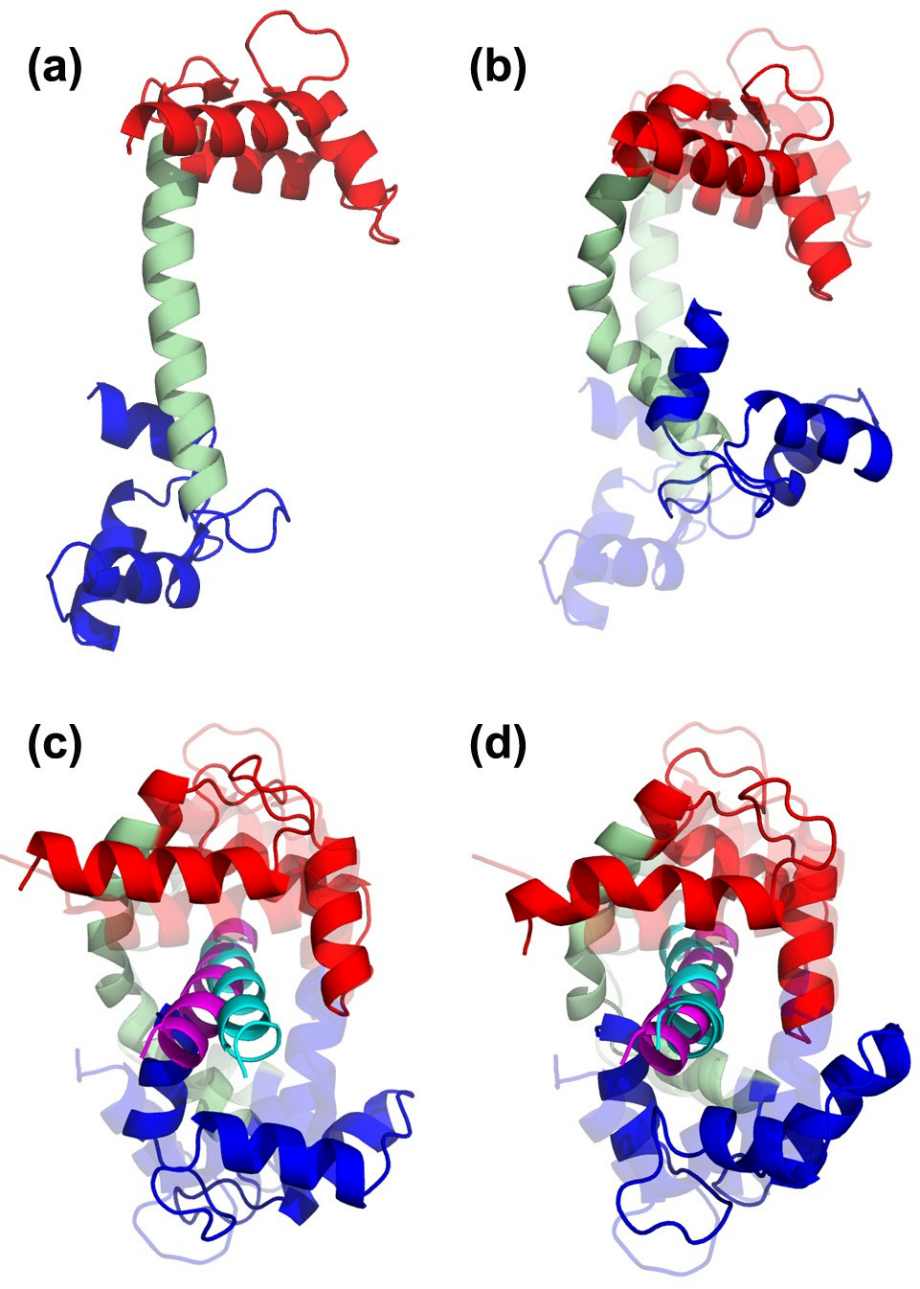
Calmodulin structures, where N-lobe, C-lobe and helical linker are shown in red, light green and blue, respectively. (a) Extended crystal structure (pdb id: 1cll) is used as the starting structure for generating unbound CAM conformers. (b) Conformer gen8 from CAM blind search is aligned to 1cll (transparent, RMSD 6.8 Å). (c) After peptide docking to gen8, the closest docking pose to bound crystal structure (1wrz, transparent) is shown (CAM with 6.8 Å, peptide with 5 Å backbone RMSD. (d) Conformer generation with bound peptide (CAM-pep) leads to further closure towards 1wrz. Here, conformer gen3 from CAM-pep is aligned on 1wrz with a complex carbon alpha RMSD of 3.7 Å (ligand backbone RMSD 2.9 Å). Magenta and cyan peptides correspond to the crystal structure and docked poses, respectively.

Calcium binding protein calmodulin functions as a multipurpose intracellular Ca^2+^ receptor that is expressed in all eukaryotic cells. CAM consists of a single polypeptide chain with four Ca^2+^ binding sites (two on each lobe). Three distinct regions in the structure consist of N-lobe, C-lobe and the long helical linker connecting them (Fig 7). Having an important role in calcium signaling pathways, it can bind to various target proteins in the cell to alter their activity, while undergoing large conformational transitions.

CAM’s transition from its fully-extended (FE) to fully-closed (FC) state is an extremely challenging one, which has not been studied by unbiased conformational searches, at least to our knowledge. We applied the generation procedure (blind and energy-based) on apo FE structure (pdb id: 1cll) in order to observe the accessibility of the FC state. As the RMSD between FE and FC states is extremely large (15 Å), we preferred to use a larger deformation RMSD (3 Å) during conformer generation. Still, at the end of eight CAM generations (Table 2), the closest conformer to the FC form has an RMSD of 6.8 Å in blind search.

In crystal structures, the FC state appears only with a ligand, such as the helical peptide in our closed reference structure (pdb id: 1wrz). This lead us to the following question: Can an induced-fit-type transition lead to the FC state after peptide binding to an intermediate state? For this purpose, we first docked the peptide to CAM and then applied a second cycle of generation with the bound peptide.

Specifically, we used the intermediate CAM conformer with 6.8 Å RMSD in peptide docking. The second best cluster (from HADDOCK) yielded a docking pose agreeable with the crystal structure (ligand backbone RMSD is 5 Å). Using this pose as the starting point, we generated new conformers with the helical peptide (for three generations with deformation of 3 Å). This “CAM-pep” conformer generation decreased the receptor RMSD to 3.8 Å (peptide backbone RMSD is 2.9 Å). As shown in Fig 7, the bound peptide seems to effectively guide the receptor towards the FC state.

## Discussion

The utility of ENM-based methodologies has been well-documented for incorporating backbone flexibility in protein-protein docking studies [3]. But in the case of small ligand docking, such methodologies have been applied to proteins undergoing relatively small conformational changes upon ligand binding [16,17,20,58,59]. In the current study, we aimed to assess the performance of our new ENM-based conformer generation method in small ligand docking, specifically for proteins undergoing large conformational changes. Such an evaluation has not been carried out so far, at least to our knowledge.

### Assessment of conformer generation procedure

We were able to obtain conformers close to the ligand-bound structure for AK (RMSD = 2.4 Å), BC (1.7 Å), LAO (1.7 Å) and DBP (1.5 Å) starting from initial RMSDs of 7.1, 4.1, 4.7 and 6.5 Å, respectively. The procedure requires only the apo structure for conformer generation, which is an advantage in the absence of information about the holo structure. As a result of the clustering, a tractable number of conformers is obtained, especially when conformers with smaller RG than the apo structure are filtered at the end of the generation procedure. If any other experimental data such as FRET distances are available, they may also be applied as filtering criteria.

We only used the first three softest modes of ENM for the generation of conformers. The reason for this choice is based on our findings [34] with an ENM-Monte Carlo hybrid technique [33], which is employed for generating transition pathways of proteins with large conformational changes. In that study, the protein generally selects the conformations along the softest modes (primarily first or second) for the transition from open to closed state. However, many more modes could in principle be needed as shown by Tama et al. [10] and Pontiggia et al. [60]. So, the number of modes in the procedure can be increased for systems of interest, which would in turn necessitate a reduction in the number of generations performed. For example, we applied the same methodology with five modes to the enzyme triosephosphate isomerase (results not shown), expressing a loop closing motion (loop RMSD of 4.5 Å) over the binding site for efficient catalysis. Two cycles were sufficient to observe loop closure in the generated conformers and produce satisfactory docking results in conformity with our previous study [61].

Strong points of this relatively simple method can be listed as follows: A reasonable number of conformers and close-to-bound ones can be produced due to clustering and post-filtering procedures. Use of implicit solvent minimization produces realistic conformers, which can be used for ensemble docking. Relatively large deformation factor and limited number of collective modes make it possible to reach the closed state in few cycles, except for the case of CAM.

CAM has especially been challenging in terms of predicting its unbiased transition from extended to fully closed (initial RMSD of 15 Å). In our work, such a transition could be realized in a two-stage conformer-generation procedure (lowest receptor RMSD of 3.8 Å), which hints at a ligand-induced transition based on a partially-closed conformer. Thus, our technique is reproducing intermediate states, as well, which may further be applicable for induced-fit transitions.

### Ligand docking to generated conformer

To assess the performance of generated conformers in docking, either an inhibitor or native ligand (peptide) was docked to the known binding site. For AK, LAO and DBP, the highest scoring and most populated cluster corresponds to the best pose with lowest ligand RMSDs of 2.9, 2.0 and 1.5 Å (Table 2), respectively. In the case of dimeric BC, the lowest ligand RMSD of 1.8 Å is observed for the most closed position of the ATP grasp domain (in the cluster with highest score). For periplasmic binding proteins (LAO and DBP) with hinge-bending motion of two domains, the best pose is obtained for the conformer, which has the smallest RG and the closest RMSD to the closed form. In contrast, an intermediate state of AK (partially closed LID and open NMP domains) yields the best pose as opposed to the closest conformer to bound structure, among the sequence of conformers. This finding is in agreement with an MD study [54] and experiments [53], both stating that this intermediate state is highly populated in the transition pathway of AK. Thus, our methodology seems to provide such significant intermediate state conformers, as well.

The intermediate conformers can be used as a starting point for holo structure modelling. In CAM case, by docking the peptide to a generated structure with an RMSD of 6.8 Å to the bound state, we obtained a pose with ligand backbone RMSD of 5 Å. Applying the method on the complex for three iterations yield close-to-bound structure with a receptor RMSD of 3.8 Å and a ligand backbone RMSD of 2.9 Å. This two-stage procedure can be used as an alternative strategy in the prediction of holo structure.

In summary, our generation technique is successful in generating both intermediate and close-to-bound conformers for multi-domain and multimeric structures, using only the apo structure, which in turn produce satisfactory results in ligand docking.

### Comparison to previous studies

We will particularly mention two studies on the prediction of holo structures in hinge-bending proteins. First one is a biased sampling procedure with tCONCOORD [50] that was applied on 10 monomeric proteins exhibiting closure of two domains. By imposing the holo structure’s RG on the tCONCOORD ensemble, global backbone RMSD of the best model to the known holo structure was found below 1.6 Å for all proteins. The dynamic domain architecture of this set, among which six are PBPs, is very similar to LAO and DBP in our study. Our procedure performs at least as good as the mentioned work in predicting the holo conformer and the ligand RMSD for PBPs, even though no matching proteins exist among the datasets.

The “conformation explorer” by Flores and Gerstein [62] is an alternative approach, again for single chains with hinge-bending motion of two domains only. Starting with the open structure, holo structures were predicted by first identifying the structural hinge, then applying Euler rotation to one of the domains about the hinge, docking of the ligand and finally applying a short MD on the complex. Among five proteins studied, the holo structures of two periplasmic proteins (again similar but not the same as our study) and the LID domain closure of AK (excluding the third domain NMP motion) were successfully predicted. BC was an unsuccessful case for bound structure prediction, although there were structures relatively close to bound form among the generated structures, but with low score. Unfortunately, the final ligand RMSDs in the complex are not available for comparison.

Another ENM-based conformational search algorithm NMSim [24] is also worth mentioning, which can produce atomistic conformers by unbiased or RG-guided simulations. Minimum RMSDs observed in the unbiased trajectories are slightly higher than those in our method (3.1 Å for AK and 2.3 Å for LAO).

### Docking to intermediate states

Our study underlines the necessity of docking to intermediate states when large hinge conformational changes are involved. Especially for transitions that are more complex than two-domain hinge bending closure, prediction of intermediates may be equally important as observing the holo conformer. In the two-stage procedure employed for CAM, induced-fit and conformational search mechanisms [63,64] complement each other for a proper approach to the holo state. Whether such a procedure in fact mimics the real process of binding, at least in certain aspects, needs further work. In as much as satisfactory binding poses have been observed for some intermediate conformers of LAO and DBP, application of the two-stage procedure to other proteins seems conceivable, which relaxes the necessity of getting close to the holo structure for a successful prediction. In other words, carrying out 3–4 cycles of conformer generation, followed by docking and further conformer generation with the bound ligand appears as a plausible alternative. For this purpose, our mixed coarse-grained approach emerges as an effective means of incorporating any type of docked ligand systematically in the ENM step [65,66].

Finally, we point to the diversity of our dataset in terms of dynamic domain decomposition/motion and inclusion of multimeric proteins. Dockings to AK and CAM present especially challenging cases in comparison to existing studies on large conformational changes.

## Methods

### A new ENM-based atomistic conformer generation algorithm

In our previous study [67], we generated realistic atomistic conformers of cyclophilin A by deforming along each softest ENM mode independently and subsequent energy minimization using implicit solvent method to eliminate steric clashes. These conformers with RMSDs up to 1.2 Å from original structure were further utilized in ensemble docking. However, such an approach was not suitable for proteins exhibiting large conformational changes between apo and bound forms.

In our current work, we devised an iterative algorithm that can be successfully applied to large conformational transitions with RMSDs above 4 Å. This is an unbiased procedure, for which the input is an apo/unbound protein structure from experiment. The steps of the algorithm are explained in detail below.

1. Starting apo crystal structure is minimized using implicit solvent model. For the minimization, AMBER [68] is used with ff03 force field parameters [69]. 500 cycles of steepest descent is followed by conjugate gradient with convergence criterion for the energy gradient set to 0.01 kcal/mol Å. Pairwise generalized Born model [70,71] is used for the minimization with implicit solvent with non-bonded interactions cut-off being 16 Å. Using a modified generalized Born theory based on the Debye-Hückel limiting law for ion screening of interactions [72], the concentration of 1–1 mobile counter-ions in solution is set to 0.1 M.
2. ENM is applied on each energetically-minimized structure. A modified version [65] of classical ENM [8,9] based on coordinates of residue centroids is used. In this version, the strength of harmonic interactions between any two residue pairs scales with the number of their interacting atom pairs that fall within a cut-off radius of 10 Å.
3. Eigenvectors ***u_j_*** of the three slowest modes extracted from ENM are linearly combined (scaled based on their frequency $\lambda_{j}^{1/2}$) using three coefficients *a_j_*:
  $$V_{i}=\sum a_{j}u_{j}/\lambda_{j}^{1/2}\;with\;a_{j}=-1,0,1$$ (1)
  and the minimized structure is deformed along the direction vectors ***d_i_*** obtained from the combination of slow modes (***V_i_***) using a deformation RMSD (*DF*) of 2 Å (3 Å for calmodulin):
  $$v_{i}=DF\times N^{0.5}\times (V_{i}/\left|V_{i}\right|)\;with\;N\;being\;the\;number\;of\;nodes$$ (2)
  The ENM deformation determined for a residue centroid is applied to all the atoms of that residue. As a result, (3^N^-1) new atomistic conformers are generated for each representative conformer.
4. Generated conformers at the current iteration, together with parent structures from previous iterations, are clustered using k-means algorithm of MMTSB toolset [73], based on mutual RMSD values. RMSD cut-off for clusters is set to *DF*.
5. Conformers with lowest RMSD to the average structure of each cluster are selected as representative conformers, excluding the cluster(s) containing any parent structure(s).
6. The above procedure, starting with Step 1, is repeated for each representative structure in subsequent stages/iterations.

In summary, the procedure starts by performing ENM on the minimized native (apo, unbound) protein structure. In consequent iterations (or generations), ENM is performed on each representative conformer from previously generated clusters. Such a procedure performs a “**blind**” conformer generation without any bias towards the bound structure, i.e. a target.

Alternatively, we perform an “**energy-based**” selection, where new representative conformers that have lower energy compared to that of minimized open/unbound protein are selected for conformer generation in the next cycle/generation. If no such conformers exist at the end of a generation, the procedure ends. The application of such a criterion, if applicable, leads to a reduction in the total number of generated conformers. In fact, comparison of receptor energy after minimization indicates lower energy for the bound conformer (without ligand) than the apo state for AK, LAO and CAM (see S1 Table).

Moreover, at the end of the blind or energy-based generations, all representative conformers are subject to an RG post-filtering criterion to further reduce the number of conformers by imposing the condition of a lower RG than the apo structure. The RG that is calculated based on Cα atoms represents the overall size of the protein, which is expected to decrease for hinge-bending proteins upon ligand binding [50].

#### Number of generations

The criterion for ending the iteration scheme in blind generation is in principal user-dependent. However, performing too many generations becomes computationally expensive (for both clustering and minimization) and also leads to the production of irreparably distorted structures, which fail to be energetically minimized. In the absence of any experimental data on the target structure, the generation scheme could be automatically terminated by imposing a maximum number of conformers, which does not always guarantee a satisfactory approach to target. An initial estimate on the degree of conformational change could also be useful in this respect, which can be based on vibrational frequencies as described in recent studies on protein-protein docking [74,75]. Alternatively, one can decide on the total number of generations based on the predicted RG value by a correlation developed for monomers [76]. This correlation may be especially useful in the absence of information on the bound state. Details on RG can be found in S1 Table and Fig 2.

#### Number of modes

We chose to use the first three modes to generate the conformers based on the findings of our recent study [34]. However, the user can consider incorporating a lower or higher number of modes of interest. S17 Table lists the overlap value (inner product) between each slowest mode’s eigenvector (based on initial structure) and the experimental transition, i.e. the apo-to-holo displacement vector. For all cases except calmodulin, the overlap value is quite high for the first mode. Thus, our iterative technique should, in principle, lead to a satisfactory approach to the intermediate and holo states by the combination of a few slowest modes.

### Docking procedures

#### Non-peptide ligands

For AK and BC dockings, both the receptor and the ligand were prepared using AutoDockTools [77]. For each receptor conformer, 10 parallel runs with 10 poses each were performed using the Lamarckian genetic algorithm of AutoDock v4.0 [77] to explore the conformational space. Each run consisted of 25x10^6^ energy evaluations. Grid box was located at the binding site of the ligand, covering receptor residues interacting with the ligand. The box dimensions were 64 x 72 x 80 Å for AK, and 80 x 80 x 80 Å for BC with 0.375 Å spacing. Results of all runs were clustered using an RMSD cut-off of 2 Å. In order to reduce the complexity of conformational search for the ligand, we kept ATP rigid in BC dockings and AP5 with 2 flexible bonds in AK dockings.

#### Peptide ligands

For peptide dockings on LAO, DBP and CAM, HADDOCK web server Easy Interface [78] was used with default parameters. Residues interacting with the ligand in the complex crystal structure were selected as active residues. We also docked peptides of LAO and DBP using Autodock v4.0, however that yielded unsuccessful docking poses.

In both non-peptide and peptide ligand dockings, we used energetically minimized structures for the receptor. In case of complex structures (holo crystal structure), the ligand was removed from the complex and the receptor was subjected to energy minimization in implicit solvent, with same parameters described in Step 1 of conformer generation technique.

## Supporting Information

S1 Table Energy and radius of gyration values for apo and holo crystal structures. (DOCX)

S2 Table AK conformers using blind search (before/after RG filtering). (DOCX)

S3 Table AK conformers using energy-based search/RG filter. (DOCX)

S4 Table LAO conformers using blind search/RG filter. (DOCX)

S5 Table LAO conformers using energy-based search/RG filter. (DOCX)

S6 Table DBP conformers using blind search/RG filter. (DOCX)

S7 Table BC (monomer A) conformers using blind/energy-based search. (DOCX)

S8 Table BC (monomer B) conformers using blind/energy-based search. (DOCX)

S9 Table Peptide-bound CAM conformers using blind search. (DOCX)

S10 Table Peptide-bound CAM conformers using energy-based search. (DOCX)

S11 Table Residues interacting with AP5 in AK docking poses. (DOCX)

S12 Table Docking results for BC (with E288K mutation). (DOCX)

S13 Table Residues interacting with ATP in BC docking poses. (DOCX)

S14 Table Residues interacting with Gly-Leu in DBP dockings and crystal structure. (DOCX)

S15 Table Docking results for LAO binding protein. (DOCX)

S16 Table Residues interacting with Lys in LAO dockings and crystal structure. (DOCX)

S17 Table Overlap between first ten global modes and apo-to-holo displacement vector. (DOCX)
