# Correction: Ligand Docking to Intermediate and Close-To-Bound Conformers Generated by an Elastic Network Model Based Algorithm for Highly Flexible Proteins

**DOI:** 10.1371/journal.pone.0160130

**Published:** 2016-07-21

**Authors:** Zeynep Kurkcuoglu, Pemra Doruker

A funder was omitted from the Financial Disclosure of the published article. The complete, correct, Financial Disclosure is: This work was partially supported by the Scientific and Technological Research Council of Turkey (TUBITAK) Project No: 113M237 and Bogazici University Scientific Research Fund (BAP) Project No: 9360. The funders had no role in study design, data collection and analysis, decision to publish, or preparation of the manuscript.
