# Supplementary material for: Ligand Docking to Intermediate and Close-To-Bound Conformers Generated by an Elastic Network Model Based Algorithm for Highly Flexible Proteins
Source: PLoS One. 2016 Jun 27;11(6):e0158063. doi: 10.1371/journal.pone.0158063 (PMC4922591; doi:10.1371/journal.pone.0158063)
Supplement: S1 Table — (DOCX) [file pone.0158063.s001.docx]

**S1 Table.** Energy and radius of gyration values for apo and holo crystal structures

| Protein | Minimization energy^a^  (kcal/mol) | | Radius of gyration- RG*^b^*  (Å) | | |
| --- | --- | --- | --- | --- | --- |
|  | apo | Holo | apo | holo | predicted*^c^* |
| AK | -7065 | -7115 | 19.4 | 16.4 | 16.9 |
| BC | -27764 | -27652 | 23.2 | 21.7 | 22.3 |
| LAO | -6826 | -6853 | 19.1 | 17.7 | 17.6 |
| DBP | -14840 | -14742 | 24.7 | 22.8 | 23.5 |
| CAM | -5685 | -5710 | 20.3 | 16.5 | 14.7 |

*^a^* The energy value obtained at the end of minimization in implicit solvent. The details of the minimization are given in Methods. The energy of the holo state is calculated without the ligand.

*^b^* RG is calculated using VMD, according to following formula:

$RG=\sqrt{\left( \sum_{i=1}^{n} w(i){(r\left( i \right)-\bar{r})}^{2} \right)/\left( \sum_{i}^{n} w(i) \right)}$

where *w(i)* and *r(i)* are the mass and the position of i^th^ atom, respectively and $\bar{r}$ is the center of mass.

*^c^* RG of monomeric proteins (*N* residues long) can be predicted using the empirical equation by Kolinski and Skolnick (1):

$RG=2.2 N^{0.38}$

**Reference**

1. Kolinski A, Skolnick J. Monte Carlo simulations of protein folding. I. Lattice model and interaction scheme. Proteins. 1994;18(4):338–52.
