# Supplementary material for: Ligand Docking to Intermediate and Close-To-Bound Conformers Generated by an Elastic Network Model Based Algorithm for Highly Flexible Proteins
Source: PLoS One. 2016 Jun 27;11(6):e0158063. doi: 10.1371/journal.pone.0158063 (PMC4922591; doi:10.1371/journal.pone.0158063)
Supplement: S2 Table — (DOCX) [file pone.0158063.s002.docx]

**S2 Table.** AK conformers using blind search **(**before/after RG filtering)

| Generation/ cycle | Total number of conformers in each cycle*^b^* | Number of conformers within specific  RMSD range to closed structure *^a^* | | | | | |
| --- | --- | --- | --- | --- | --- | --- | --- |
|  |  | 2-3 Å | 3-4 Å | 4-5 Å | 5-6 Å | 6-7.2 Å | >7.2 Å*^b^* |
| 1 | 3/1 | 0 | 0 | 0 | 0 | 1 | 2/ 0 |
| 2 | 10/3 | 0 | 0 | 0 | 2 | 1 | 7/ 0 |
| 3 | 18/8 | 0 | 0 | 4 | 1 | 3 | 10/ 0 |
| 4 | 35/17 | 0 | 4 | 2 | 4 | 3 | 22/ 4 |
| 5 | 55/25 | 1 | 4 | 4 | 0 | 10 | 36/ 6 |
| 6 | 102/51 | 3 | 3 | 9 | 9 | 10 | 68/ 17 |
| 7 | 149/80 | 2 | 10 | 9 | 12 | 17 | 99/ 30 |
| All cycles | 372/185 | 6 | 21 | 28 | 28 | 45 | 244/ 57 |

*^a^* The distinct conformers generated at each cycle are classified according to their alpha carbon RMSD with respect to the closed (ligand-bound) crystal structure. As the number of iterations increases, structures closer to closed structure are obtained but the total number of generated conformers also increases.

*^b^* RG of the apo structure can be used as an additional post-filtering criterion in order to eliminate conformers with larger RGs. The second number in the column gives the number of conformers remaining after application of the RG filter. As a result, outliers that lie further away from open structure, i.e. with large RMSDs, are eliminated.
