# Supplementary material for: Ligand Docking to Intermediate and Close-To-Bound Conformers Generated by an Elastic Network Model Based Algorithm for Highly Flexible Proteins
Source: PLoS One. 2016 Jun 27;11(6):e0158063. doi: 10.1371/journal.pone.0158063 (PMC4922591; doi:10.1371/journal.pone.0158063)
Supplement: S3 Table — (DOCX) [file pone.0158063.s003.docx]

**S3 Table.** AK conformers using energy-based search**/**RG filter

| Generation/  cycle | Total number of conformers in each cycle | Number of conformers within specific  RMSD range to closed structure | | | | | |
| --- | --- | --- | --- | --- | --- | --- | --- |
|  |  | 2-3 Å | 3-4 Å | 4-5 Å | 5-6 Å | 6-7.2 Å | >7.2 Å |
| 1 | 3/1 | 0 | 0 | 0 | 0 | 1 | 2/0 |
| 2 | 9/3 | 0 | 0 | 0 | 2 | 1 | 6/0 |
| 3 | 9/5 | 0 | 0 | 3 | 0 | 2 | 4/0 |
| 4 | 13/10 | 0 | 1 | 3 | 1 | 4 | 4/1 |
| 5 | 22/19 | 1 | 2 | 5 | 3 | 3 | 8/5 |
| 6 | 27/23 | 2 | 4 | 6 | 3 | 5 | 7/3 |
| 7 | 38/31 | 1 | 6 | 6 | 6 | 7 | 12/5 |
| All cycles | 121/92 | 4 | 13 | 23 | 15 | 23 | 43/14 |
