# Supplementary material for: Ligand Docking to Intermediate and Close-To-Bound Conformers Generated by an Elastic Network Model Based Algorithm for Highly Flexible Proteins
Source: PLoS One. 2016 Jun 27;11(6):e0158063. doi: 10.1371/journal.pone.0158063 (PMC4922591; doi:10.1371/journal.pone.0158063)
Supplement: S4 Table — (DOCX) [file pone.0158063.s004.docx]

**S4 Table.** LAO conformers using blind search/RG filter

| Generation/ cycle | Total number of conformers in each cycle | Number of conformers within specific  RMSD range to closed structure | | | |
| --- | --- | --- | --- | --- | --- |
|  |  | 1-2 Å | 2-3 Å | 3-4.7 Å | >4.7 Å |
| 1 | 4/1 | 0 | 0 | 0 | 4/1 |
| 2 | 8/2 | 0 | 0 | 0 | 8/2 |
| 3 | 17/4 | 0 | 0 | 1 | 16/3 |
| 4 | 26/5 | 0 | 0 | 3 | 23/2 |
| 5 | 45/13 | 0 | 1 | 7 | 37/5 |
| 6 | 75/27 | 0 | 3 | 13 | 59/11 |
| 7 | 114/32 | 0 | 3 | 15 | 96/14 |
| All cycles | 289/84 | 0 | 7 | 39 | 243/38 |
