# Supplementary material for: Ligand Docking to Intermediate and Close-To-Bound Conformers Generated by an Elastic Network Model Based Algorithm for Highly Flexible Proteins
Source: PLoS One. 2016 Jun 27;11(6):e0158063. doi: 10.1371/journal.pone.0158063 (PMC4922591; doi:10.1371/journal.pone.0158063)
Supplement: S5 Table — (DOCX) [file pone.0158063.s005.docx]

**S5 Table.** LAO conformers using energy-based search/RG filter

| Generation/ cycle | Total number of conformers in each cycle | Number of conformers within specific  RMSD range to closed structure | | | |
| --- | --- | --- | --- | --- | --- |
|  |  | 1-2 Å | 2-3 Å | 3-4.7 Å | >4.7 Å |
| 1 | 2/0 | 0 | 0 | 0 | 2/0 |
| 2 | 3/0 | 0 | 0 | 0 | 3/0 |
| 3 | 6/2 | 0 | 0 | 1 | 5/1 |
| 4 | 3/1 | 0 | 0 | 1 | 2/0 |
| 5 | 3/2 | 0 | 1 | 1 | 1/0 |
| 6 | 4/3 | 1 | 2 | 0 | 1/0 |
| 7 | 0/0 | 0 | 0 | 0 | 0/0 |
| All cycles | 21/8 | 1 | 3 | 3 | 14/1 |
