# Supplementary material for: Ligand Docking to Intermediate and Close-To-Bound Conformers Generated by an Elastic Network Model Based Algorithm for Highly Flexible Proteins
Source: PLoS One. 2016 Jun 27;11(6):e0158063. doi: 10.1371/journal.pone.0158063 (PMC4922591; doi:10.1371/journal.pone.0158063)
Supplement: S6 Table — (DOCX) [file pone.0158063.s006.docx]

**S6 Table.** DBP conformers using blind search/RG filter

| Generation/ cycle | Total number of conformers in each cycle | Number of conformers within specific  RMSD range to closed structure | | | | | |
| --- | --- | --- | --- | --- | --- | --- | --- |
|  |  | 1-2 Å | 2-3 Å | 3-4 Å | 4-5 Å | 5-6.5 Å | >6.5 Å |
| 1 | 3/1 | 0 | 0 | 0 | 1 | 0 | 2/0 |
| 2 | 8/3 | 0 | 0 | 0 | 1 | 2 | 5/0 |
| 3 | 17/9 | 0 | 1 | 1 | 1 | 4 | 10/2 |
| 4 | 24/6 | 0 | 1 | 2 | 0 | 4 | 17/0 |
| 5 | 48/18 | 2 | 0 | 4 | 1 | 5 | 36/7 |
| All cycles | 100/37 | 2 | 2 | 7 | 4 | 15 | 70/9 |
