# Supplementary material for: Ligand Docking to Intermediate and Close-To-Bound Conformers Generated by an Elastic Network Model Based Algorithm for Highly Flexible Proteins
Source: PLoS One. 2016 Jun 27;11(6):e0158063. doi: 10.1371/journal.pone.0158063 (PMC4922591; doi:10.1371/journal.pone.0158063)
Supplement: S7 Table — (DOCX) [file pone.0158063.s007.docx]

**S7 Table.** BC (monomer A) conformers using blind/energy-based search

| Generation/ cycle | Total number of conformers in each cycle | Number of conformers within specific  RMSD range to closed structure | | | |
| --- | --- | --- | --- | --- | --- |
|  |  | 1-2 Å | 2-3 Å | 3-4.1 Å | >4.1 Å |
| 1 | 3/0 | 0 | 0 | 0 | 3/0 |
| 2 | 7/1 | 0 | 0 | 1 | 6/0 |
| 3 | 8/4 | 0 | 2 | 1 | 5/1 |
| 4 | 17/7 | 0 | 3 | 2 | 12/2 |
| All cycles | 35/12 | 0 | 5 | 4 | 26/3 |
