# Supplementary material for: Ligand Docking to Intermediate and Close-To-Bound Conformers Generated by an Elastic Network Model Based Algorithm for Highly Flexible Proteins
Source: PLoS One. 2016 Jun 27;11(6):e0158063. doi: 10.1371/journal.pone.0158063 (PMC4922591; doi:10.1371/journal.pone.0158063)
Supplement: S8 Table — (DOCX) [file pone.0158063.s008.docx]

**S8 Table.** BC (monomer B) conformers using blind/energy-based search

| Generation/ cycle | Total number of conformers in each cycle | Number of conformers within specific  RMSD range to closed structure | | | |
| --- | --- | --- | --- | --- | --- |
|  |  | 1-2 Å | 2-3 Å | 3-4.1 Å | >4.1 Å |
| 1 | 3/2 | 0 | 0 | 1 | 2/1 |
| 2 | 7/3 | 0 | 1 | 1 | 5/1 |
| 3 | 8/1 | 0 | 1 | 0 | 7/0 |
| 4 | 17/5 | 3 | 0 | 2 | 12/0 |
| All cycles | 35/11 | 3 | 2 | 4 | 26/2 |
