# Supplementary material for: Ligand Docking to Intermediate and Close-To-Bound Conformers Generated by an Elastic Network Model Based Algorithm for Highly Flexible Proteins
Source: PLoS One. 2016 Jun 27;11(6):e0158063. doi: 10.1371/journal.pone.0158063 (PMC4922591; doi:10.1371/journal.pone.0158063)
Supplement: S9 Table — (DOCX) [file pone.0158063.s009.docx]

**S9 Table.** Peptide-bound CAM conformers using blind search

| Generation/ cycle | Total number of conformers in each cycle | Number of conformers within specific  RMSD range to closed structure*^a^* | | | | |
| --- | --- | --- | --- | --- | --- | --- |
|  |  | 3-4 Å | 4-5 Å | 5-6 Å | 6-6.7 Å | >6.7 Å |
| 1 | 4 | 0 | 0 | 1 | 1 | 2 |
| 2 | 9 | 0 | 1 | 0 | 0 | 8 |
| 3 | 19 | 1 | 2 | 2 | 3 | 11 |
| All cycles | 32 | 1 | 3 | 3 | 4 | 21 |

*^a^* RMSD includes protein and peptide alpha-carbons.
