# Supplementary material for: Ligand Docking to Intermediate and Close-To-Bound Conformers Generated by an Elastic Network Model Based Algorithm for Highly Flexible Proteins
Source: PLoS One. 2016 Jun 27;11(6):e0158063. doi: 10.1371/journal.pone.0158063 (PMC4922591; doi:10.1371/journal.pone.0158063)
Supplement: S10 Table — (DOCX) [file pone.0158063.s010.docx]

**S10 Table.** Peptide-bound CAM conformers using energy-based search

| Generation/ cycle | Total number of conformers in each cycle | Number of conformers within specific  RMSD range to closed structure | | | | |
| --- | --- | --- | --- | --- | --- | --- |
|  |  | 3-4 Å | 4-5 Å | 5-6 Å | 6-6.7 Å | >6.7 Å |
| 1 | 4 | 0 | 0 | 1 | 1 | 2 |
| 2 | 7 | 0 | 2 | 0 | 1 | 4 |
| 3 | 8 | 1 | 0 | 1 | 1 | 5 |
| All cycles | 19 | 1 | 2 | 2 | 3 | 11 |

*^a^* RMSD includes protein and peptide alpha-carbons.
