# Supplementary material for: Ligand Docking to Intermediate and Close-To-Bound Conformers Generated by an Elastic Network Model Based Algorithm for Highly Flexible Proteins
Source: PLoS One. 2016 Jun 27;11(6):e0158063. doi: 10.1371/journal.pone.0158063 (PMC4922591; doi:10.1371/journal.pone.0158063)
Supplement: S11 Table — (DOCX) [file pone.0158063.s011.docx]

**S11 Table.** Residues interacting with AP5 in AK docking poses

|  | Residues within 4.5 Å of the ligand*^a^* | |
| --- | --- | --- |
| Generation/cycle | Residues common with crystal structure | Additional residues |
| 1ake (complex) | P9, G10, G12, K13, G14, T15, T31, R36, M53, K57, V59, G85, R88, Q92, R123, Y133, R156, R167, K200 |  |
| Apo | P9, G10, R123, R167 | K166, G170 |
| gen1 | R123, R167 | N138, G170 |
| gen2 | G10, G12, R123, R156, R167 | D158 |
| gen3 | P9, G10, G12, K13, R123, R167 | D84 |
| **gen4** | G10, G12, K13, G14, R36, R123, R167, K200 | S30, G32 |
| gen5 | R36, K57, R123, R167 | A8 |
| gen6 | R36, M53, R156 | K40, D54, K157 |
| gen7 | P9, R36, R123, R167 | Q18, R119, I120, N138, D158 |

*^a^* These residues belong to different domains, namely the CORE (residues 1-29, 60-121, 160-214), the LID (residues 122-159) and the NMP (residues 30-59) domains.
