# Supplementary material for: Ligand Docking to Intermediate and Close-To-Bound Conformers Generated by an Elastic Network Model Based Algorithm for Highly Flexible Proteins
Source: PLoS One. 2016 Jun 27;11(6):e0158063. doi: 10.1371/journal.pone.0158063 (PMC4922591; doi:10.1371/journal.pone.0158063)
Supplement: S12 Table — (DOCX) [file pone.0158063.s012.docx]

**S12 Table.** Docking results for BC (with E288K mutation)

|  | RMSD (Å) to 1dv2 (monomer/dimer) | **Conformer clusters** | **Binding energy (kcal/mol)** | **Number of elements/poses** | **Ligand RMSD Å** |
| --- | --- | --- | --- | --- | --- |
| **1dv2** | 0.0/0.0 | Cluster 1 | -8.31 | 18 | 0.8 |
|  |  | Cluster 2 | -5.68 | 17 | 14 |
|  |  | Cluster 3 | -5.63 | 10 | 13 |
|  |  | Cluster 4 | -5.63 | 34 | 12 |
|  |  | Cluster 5 | -5.62 | 17 | 12 |
|  |  | Cluster 6 | -5.5 | 4 | 14 |
| **Apo** | 4.1/4.6 | Cluster 1 | -3.98 | 24 | 11 |
|  |  | Cluster 2 | -3.78 | 11 | 12 |
|  |  | Cluster 3 | -3.76 | 8 | 6.7 |
|  |  | Cluster 4 | -3.63 | 57 | 5.3 |
| **Gen 1** | 4.2 /5.7 | Cluster 1 | -4.43 | 26 | 11 |
|  |  | Cluster 2 | -3.93 | 14 | 12 |
|  |  | Cluster 3 | -3.84 | 60 | 5.5 |
| **Gen 2** | 3.2/5.3 | Cluster 1 | -3.10 | 78 | 15 |
|  |  | Cluster 2 | -2.72 | 4 | 15 |
|  |  | Cluster 3 | -2.44 | 2 | 13 |
|  |  | Cluster 4  Cluster 5 | -2.42  -2.32 | 2  14 | 16  6.9 |
| **Gen 3** | 2.3/6.1 | Cluster 1 | -4.57 | 40 | 12 |
|  |  | Cluster 2 | -4.30 | 58 | 9.4 |
|  |  | Cluster 3 | -4.16 | 1 | 14 |
|  |  | Cluster 4 | -4.08 | 1 | 6.7 |
| **Gen 4** | 1.9/5.3 | Cluster 1 | -4.53 | 28 | 1.8 |
|  |  | Cluster 2 | -4.33 | 67 | 7.4 |
|  |  | Cluster 3 | -4.23 | 5 | 14 |
