# Supplementary material for: Ligand Docking to Intermediate and Close-To-Bound Conformers Generated by an Elastic Network Model Based Algorithm for Highly Flexible Proteins
Source: PLoS One. 2016 Jun 27;11(6):e0158063. doi: 10.1371/journal.pone.0158063 (PMC4922591; doi:10.1371/journal.pone.0158063)
Supplement: S13 Table — (DOCX) [file pone.0158063.s013.docx]

**S13 Table.** Residues interacting with ATP in BC docking poses

|  | Residues within 4.5 Å of the ligand | |
| --- | --- | --- |
| Generation/cycle | Residues common with crystal structure | Additional residues |
| 1dv2 (crystal) | K116, K159, G165, G166, M169, E201, K202, L204, H236, L278, K288 |  |
| 1dv2 Cluster 1 (docked) | K116, K159, G165, M169, E201, K202, L204, H236, L278, K288 |  |
| Apo Cluster 4 | K116, H236, L278, K288 | E87, G114, I287, I437 |
| gen1 Cluster 3 | K116, H236, L278, K288 | E87, G114, I287, I437 |
| **gen4 Cluster 1** | K159, E201, K202, L204, H236, L278, K288 | V131, Y203, H209, Q233, I287, I437 |
