# Supplementary material for: Ligand Docking to Intermediate and Close-To-Bound Conformers Generated by an Elastic Network Model Based Algorithm for Highly Flexible Proteins
Source: PLoS One. 2016 Jun 27;11(6):e0158063. doi: 10.1371/journal.pone.0158063 (PMC4922591; doi:10.1371/journal.pone.0158063)
Supplement: S14 Table — (DOCX) [file pone.0158063.s014.docx]

**S14 Table.** Residues interacting with Gly-Leu in DBP dockings and crystal structure

|  | Residues within 4.5 Å of the ligand | |
| --- | --- | --- |
| Generation/cycle | Residues common with crystal structure | Additional residues |
| Complex (1dpp) | T20, S21, G22, Y114, R355, Y357, W405, T406, D408 |  |
| Apo | Y114, R355, Y357, W405, T406, D408 | P356, G404, G407 |
| Gen1 | Y114, R355, Y357, W405, T406, D408 | P356, W386, L390, M403, G404 |
| Gen3 | Y114, R355, Y357, W405, T406, D408 | M403, G404, Y431 |
| Gen5 | I20, S21, G22, Y114, R355, Y357, W405, T406, D408 | T23, Y25, L390, M403, G404, Y431 |
