# Supplementary material for: Ligand Docking to Intermediate and Close-To-Bound Conformers Generated by an Elastic Network Model Based Algorithm for Highly Flexible Proteins
Source: PLoS One. 2016 Jun 27;11(6):e0158063. doi: 10.1371/journal.pone.0158063 (PMC4922591; doi:10.1371/journal.pone.0158063)
Supplement: S15 Table — (DOCX) [file pone.0158063.s015.docx]

**S15 Table.** Docking results for LAO binding protein

|  | RMSD  (C-alpha) to 1lst Å | Conformer clusters* | HADDOCK score | Number of elements | Ligand heavy atom RMSD Å |
| --- | --- | --- | --- | --- | --- |
| 1lst | 0.0 | 1 | -39.5±1.7 | 179 | 1.5 |
|  |  | 2 | -6.3±7.3 | 12 | 14 |
|  |  | 3 | 4.5±7.4 | 5 | 12 |
| Apo | 4.7 | 2 | -28.6±1.8 | 41 | 4.2 |
|  |  | 3 | -28.3±2.0 | 37 | 4.0 |
|  |  | 4 | -28.1±2.5 | 31 | 5.0 |
|  |  | 6 | -27.3±3.9 | 14 | 2.4 |
|  |  | 1 | -25.8±1.5 | 48 | 4.4 |
|  |  | 5 | -24.3±6.5 | 15 | 3.7 |
|  |  | 7 | -18.7±4.9 | 9 | 4.9 |
|  |  | 8 | -16.1±3.8 | 5 | 5.0 |
| Gen 1 | 6.4 | 4 | -33.5±4.8 | 30 | 5.6 |
|  |  | 3 | -26.0±1.1 | 35 | 4.8 |
|  |  | 1 | -25.2±2.2 | 46 | 5.7 |
|  |  | 5 | -24.4±2.7 | 22 | 6.1 |
|  |  | 2 | -23.0±2.4 | 41 | 6.3 |
|  |  | 6 | -17.6±6.0 | 11 | 5.2 |
|  |  | 7 | -8.4±2.9 | 7 | 5.5 |
|  |  | 8 | -8.3±7.0 | 5 | 5.1 |
| Gen 2 | 7.0 | 1 | -33.5±6.1 | 46 | 8.3 |
|  |  | 5 | -31.6±1.8 | 18 | 5.7 |
|  |  | 2 | -30.3±1.5 | 39 | 5.4 |
|  |  | 3 | -29.8±4.6 | 38 | 5.3 |
|  |  | 4 | -29.1±1.7 | 28 | 5.2 |
|  |  | 8 | -29.1±9.6 | 6 | 5.6 |
|  |  | 7 | -25.9±3.4 | 9 | 4.3 |
|  |  | 6 | -19.8±4.1 | 13 | 4.9 |
| Gen 3 | 5.3 | 2 | -38.1±6.7 | 43 | 4.4 |
|  |  | 1 | -33.6±4.6 | 50 | 5.4 |
|  |  | 3 | -33.3±2.7 | 32 | 4.5 |
|  |  | 4 | -31.8±3.2 | 29 | 4.4 |
|  |  | 7 | -27.8±2.0 | 13 | 4.3 |
|  |  | 6 | -27.6±4.3 | 14 | 4.0 |
|  |  | 5 | -26.1±4.1 | 14 | 4.3 |
|  |  | 8 | -9.5±5.5 | 4 | 4.2 |
| Gen 4 | 3.8 | 2 | -37.2±0.9 | 78 | 3.3 |
|  |  | 1 | -37.2±0.6 | 116 | 2.1 |
|  |  | 3 | -21.7±6.8 | 5 | 3.4 |
| Gen 5 | 2.7 | 1 | -46.2±0.7 | 200 | 2.2 |
| Gen 6 | 1.7 | 1 | -48.5±1.8 | 122 | 2.0 |
|  |  | 2 | -47.4±2.3 | 67 | 1.9 |
|  |  | 3 | -29.0±6.4 | 7 | 5.5 |

*HADDOCK names the most crowded cluster as “Cluster 1”.
