# Supplementary material for: Ligand Docking to Intermediate and Close-To-Bound Conformers Generated by an Elastic Network Model Based Algorithm for Highly Flexible Proteins
Source: PLoS One. 2016 Jun 27;11(6):e0158063. doi: 10.1371/journal.pone.0158063 (PMC4922591; doi:10.1371/journal.pone.0158063)
Supplement: S16 Table — (DOCX) [file pone.0158063.s016.docx]

**S16 Table.** Residues interacting with Lys in LAO dockings and crystal structure

|  | Residues within 4.5 Å of the ligand | |
| --- | --- | --- |
| Generation/cycle | Residues common with crystal structure | Additional residues |
| 1lst (complex) | D11, Y14, S69, S70, S72, R77, S120, T121, D161 |  |
| Apo cluster 6 | Y14, S70, S72, T121, D161 | A90 |
| **gen4** | D11, S69, S72, T121, D161 | A90 |
| gen6 | D11, S69, S70, S72, R77 | T13, G119 |
