# Supplementary material for: Ligand Docking to Intermediate and Close-To-Bound Conformers Generated by an Elastic Network Model Based Algorithm for Highly Flexible Proteins
Source: PLoS One. 2016 Jun 27;11(6):e0158063. doi: 10.1371/journal.pone.0158063 (PMC4922591; doi:10.1371/journal.pone.0158063)
Supplement: S17 Table — (DOCX) [file pone.0158063.s017.docx]

**S17 Table.** Overlap between first ten global modes and apo-to-holo displacement vector

| **Modes** | **AK** | **BC** | **CAM** | **DBP** | **LAO** |
| --- | --- | --- | --- | --- | --- |
| **1** | 0.7459 | 0.5880 | 0.1345 | 0.8866 | 0.8167 |
| **2** | 0.1302 | 0.0855 | 0.2880 | 0.3189 | 0.4671 |
| **3** | 0.1089 | 0.0871 | 0.3863 | 0.1260 | 0.0999 |
| **4** | 0.2525 | 0.2979 | 0.3312 | 0.1070 | 0.0557 |
| **5** | 0.2404 | 0.3681 | 0.0985 | 0.1291 | 0.0025 |
| **6** | 0.0230 | 0.1535 | 0.3901 | 0.0208 | 0.0142 |
| **7** | 0.0768 | 0.2250 | 0.1318 | 0.0580 | 0.0602 |
| **8** | 0.1833 | 0.0586 | 0.0990 | 0.0247 | 0.1752 |
| **9** | 0.0365 | 0.0213 | 0.0064 | 0.0369 | 0.0291 |
| **10** | 0.0159 | 0.0263 | 0.0571 | 0.0241 | 0.0538 |
